# Fast Synchronization Scheme Using 2-Way Parallel Rendezvous in IEEE 802.15.4 TSCH †

**DOI:** 10.3390/s20051303

**Published:** 2020-02-27

**Authors:** Byeong-Hwan Bae, Sang-Hwa Chung

**Affiliations:** Department of Electrical, Electronics, and Computer Engineering, Pusan National University, Busan 46241, Korea; bbh2002@pusan.ac.kr

**Keywords:** IEEE 802.15.4, parallel rendezvous, fast synchronization, time-slotted channel hopping (TSCH)

## Abstract

The high level of robustness and reliability required in industrial environments can be achieved using time-slotted channel hopping (TSCH) medium access control (MAC) specified in institute of electrical and electronics engineers (IEEE) 802.15.4. Using frequency channel hopping in the existing TSCH network, a parallel rendezvous technique is used to exchange packets containing channel information before network synchronization, thereby facilitating fast network synchronization. In this study, we propose a distributed radio listening (DRL)–TSCH technique that uses a two-way transmission strategy based on the parallel rendezvous technique to divide the listening channel by sharing the channel information between nodes before synchronization. The performance evaluation was conducted using the OpenWSN stack, and the actual experiment was carried out by utilizing the OpenMote-cc2538 module. The time taken for synchronization and the number of rendezvous packets transmitted were measured in linear and mesh topologies, and the amount of energy used was evaluated. The performance results demonstrate a maximum average reduction in synchronization time of 67% and a reduction in energy consumption of 58% when compared to the performance results of other techniques.

## 1. Introduction

**Figure 1:**
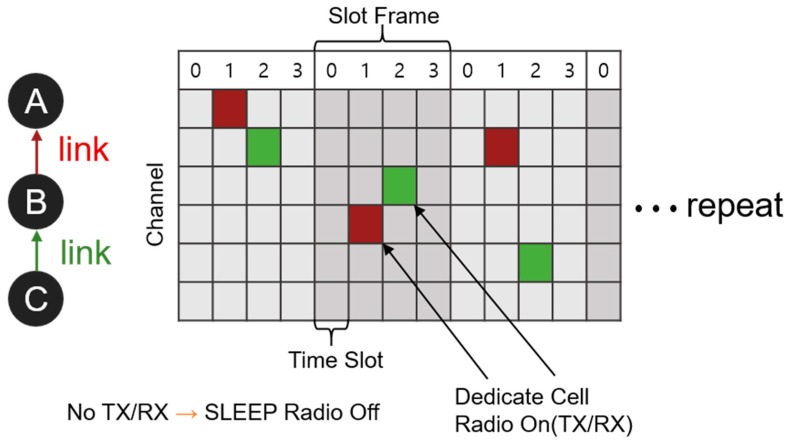
TSCH Operation.

In 2012, the IEEE announced a new IEEE 802.15.4e standard [1] that extends existing IEEE 802.15.4 [2] medium access control (MAC) functionality to address the needs of industrial applications and included it in IEEE 802.15.4 (2015) [3]. TSCH combines timeslot access with multi-channel and channel hopping technology to ensure high reliability and low power. This permits existing WirelessHART [4] and ISA100.11a [5] standards for industrial radio standards that meet the requirements of industrial applications to use TSCH. The IETF 6TiSCH Working Group [6] also standardizes the combination of efficiency and ease of use of IPv6 and employs the IEEE 802.15.4 TSCH mode as the MAC stack. In Internet of Things (IoT) environments, nodes are usually battery operated and energy-limited, and therefore, energy consumption issues are important. Nodes synchronized using TSCH reduce energy consumption through radio duty cycle (RDC) operation based on time division multiple access (TDMA). In addition, a dedicated cell can be allocated and transmitted without interference between devices. In TSCH, data can be transmitted after synchronization. Data transmission is performed in units of timeslots as shown in Figure 1. A slot frame is composed of a bundle of timeslots, and a link is defined as a slot having a combination of time and channel offsets, which can be used by a node for communication. A link can be shared by two or more nodes in a shared cell, in which case contention is resolved using carrier-sense multiple access (CSMA). All nodes participating in the network cycle through the scheduled slot frame repeatedly and continuously, performing one of the following operations—transmit, receive, or sleep—in the timeslot. They thereby reduce the RDC that consumes most of the energy used during the process of communication. TSCH switches channels by frequency hopping up to 16 channels per time slot. This is robust enough to counter the multipath fading that occurs frequently in industrial applications.

This study focuses on the network formation process of TSCH. TSCH creates a globally synchronized mesh network based on a coordinator, also known as a sink. The network’s formation is initiated when the coordinator transmits an enhanced beacon (EB) frame containing the information element (IE) required for the connection to advertise the existence of the network. The IEEE 802.15.4 TSCH standard does not propose advertising strategies related to EB and does not indicate which channel should send a message at what time, or at what rate. The process of forming a TSCH network is very important in terms of energy consumption and immediate data transfer requirements. The node attempting to connect to the network scans for available channels and attempts to detect the presence of the network. Because it tends to use up to 16 channels, the synchronization time becomes longer owing to the mismatch between the channel of the node transmitting the EB and the node receiving it. The situation worsens because, when longer hops comprise the network, the nodes that make up the previous hop must synchronize first. The unsynchronized node is listening for the EB with the radio continuously turned on, at which point the RDC is 100%. Therefore, the slower the network participation, the higher is the energy consumption. In the TSCH configuration proposed by RFC8180 [7], a slot frame having 101 timeslots, including a single shared timeslot, is considered. Considering that the RDC is approximately 1%, the energy consumed before joining the network is very large. Before exchanging packets at higher layers, nodes must form a TSCH network at the MAC level.

In [8], the author periodically transmits a packet called the RB (rendezvous beacon) containing channel information prior to TSCH synchronization to permit asynchronous nodes to announce their channel information, thereby contributing to fast synchronization. This is known as the parallel rendezvous strategy in [9,10]. Collecting information from neighboring nodes by sending and receiving data before synchronization is a very attractive option because the RDC is already at 100% and the additional energy consumption overhead is low. In this study, we form a rendezvous chain before synchronization based on the parallel rendezvous strategy. Each rendezvous chain provides channel information between two nodes through bidirectional packet transmission. Furthermore, we propose a method of subdividing the range of the channels scanned for EB reception. The rendezvous chain is defined as a two-way transmission and response connection that knows the channel index, channel sequence, and node ID information of every other element in the chain before TSCH network synchronization. Thus, we propose the formation of a rendezvous chain that listens for the signal by dividing the total EB scan channel range into separate receiving channels. Using this strategy, we increase the probability of receiving the EB of the entire rendezvous chain, thus facilitating the rapid network participation of the nodes that form the rendezvous chain. Therefore, this study makes four contributions to the relevant field of research.

- Our proposal permits a fast synchronization of the network, thereby reducing the energy consumed until the network synchronization of each node.
- Further, our proposal reduces network formation time and helps provide immediate access to data.
- DRL–TSCH, the proposed technique, includes four techniques, namely (1) two-way transmission strategy (2) distributed radio listening through two-way connections (3) hopping channel calculation method (4) ERB packet transmission.
- The experiment, which utilizes 10 and 25 openmote-cc2538, compares various techniques to evaluate synchronization speed, energy consumption, and pre-synchronization traffic.

This can provide fast resynchronization times for mobile workers who represent multiple nodes, and in environments with a frequent powering off and on of nodes. Section 2 deals with the standard TSCH network synchronization, various EB scheduling algorithms, and parallel research works studying the rendezvous concept. Section 3 describes the detailed method of DRL–TSCH, which is presented in this study. In Section 4, we evaluate the experimental results using real-world experiments with nine-hop linear topologies and standardized 5 × 5 grid mesh topologies. Finally, Section 5 discusses conclusions and future work. This paper is an expanded version of the previous [11], and it provides a two-way strategy, the specifics of the DRL–TSCH, the proposed technique, and additional materialization techniques. Additionally, the actual materialization using the openmote-cc2538 took place to evaluate multiple techniques in terms of synchronization speed, traffic, and energy consumption.

## 2. Background

The IEEE 802.15.4 TSCH standard does not define how to schedule an EB. Several EB scheduling algorithms contribute to fast TSCH network synchronization. EB scheduling schemes provide an efficient means to distribute the EB to multiple channels and to determine the transmission time when many nodes need to be synchronized. The parallel rendezvous scheme implements an information sharing strategy between unsynchronized nodes that does not conflict with the EB scheduling method. Therefore, when used together, faster synchronization can be achieved.

### 2.1. TSCH Synchronization

The passive scan (PS) algorithm is a basic network synchronization method defined in the IEEE802.15.4 standard. The TSCH network transmits an EB containing all the information needed by the coordinator node to synchronize with another node, thus facilitating network synchronization. To begin with, the network configuration is initiated with one coordinator. When another node receives the EB transmitted by the coordinator, TSCH synchronization of the receiver node is performed. The receiver node, now synchronized with the network can transmit the EB, thereby expanding the network. The asynchronous node starts at any point in the available channel array V and listens for the signal on that channel for one scan period. After the scan cycle, the next channel of V is selected in a round-robin fashion. The packet transmission selects a channel through Equation (1).

Channel = V [(ASN + Channel_Offset_) mod N_Ch_], (1)

**Figure 2:**
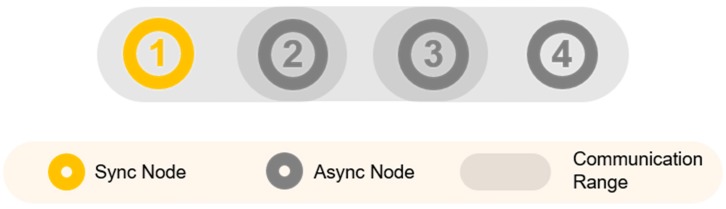
3-hop Linear Topology.

In Equation (1), ASN is an absolute slot number that is incremented at every slot when the network starts to form. The Channel Offset is used to determine the channel to be logically used in Equation (1). N_Ch_ represents the number of channels of V. The EB is broadcast from the shared slot, and the receiving node is synchronized without transmitting the ACK. Until synchronization, the node continues the scanning process until it receives an EB for the preconfigured scan cycles and the configured channel arrays. The scan period may be the same as the slot length or may be set differently. At this time, a delay occurs because of the channel mismatch between the node sending the EB and the scanning node. In addition, assuming that nodes start searching almost simultaneously because only synchronized nodes can transmit EBs, in linear topology, the time required for all nodes to connect to the TSCH network increases with the number of hops. Figure 2 displays a three-hop linear topology. In this case, node 4 may receive and synchronize the EBs transmitted by the node 3 only after node 2 and node 3 have completed synchronization.

### 2.2. EB Scheduling Algorithm

In [12], the author proposed subdividing the slot frame into the advertisement (ADV) plane and the communication plane and increasing the number of ADV slots for EB transmission. Increasing the number of ADV slots for EB transmission increases the speed of synchronization, but increases the energy consumed during transmission and reception owing to more EB slots. Therefore, the technique of [12] exhibits an increase in energy and slot consumption, both of which are limited resources in TSCH. In [13,14], the author proposed four strategies to reduce the time required to connect to a network. He proposed random vertical (RV) filling, which is an algorithm for transmitting EB by selecting one of the channel offsets, and random horizontal (RH) filling, which selects one of the multi slot frames and transmits the EB. Furthermore, enhanced coordinated vertical (ECV) filling and enhanced coordinated horizontal (ECH) filling are proposed in subsequent studies. The RV algorithm randomly selects and transmits on one of several channels in a slot capable of sending an EB; the RH algorithm randomly selects one of several slot frames and transmits in the same manner. The ECV and ECH algorithms are enhanced by utilizing the coordinator node when power is supplied from the existing RV and RH algorithms. However, in these four approaches, the synchronization time of unsynchronized nodes is greatly affected by the number of synchronized nodes. In [15,16], the authors proposed an analytical model of the network formation process in the TSCH network based on the discrete time Markov chain. In [15], the author measured the time that a new node takes to joins the network when there are already N nodes advertising on the network. In [16], a model beacon scheduling (MBS) algorithm was proposed that allowed network nodes to autonomously select the link to use for EB advertising, with the optimal EB schedule provided by the model.

In [17,18] the EB scheduling technique is proposed using the IPv6 routing protocol for low-power and lossy networks (RPL) trickle timer-based EB transmission mechanism. The author of [17] proposes a “custom trickle timer” that dynamically adjusts the EB transmission interval. The proposed technique reduces control message traffic compared to methods with a fixed short EB transmission interval but shows similar synchronization times. The author of [18] points out that the mechanism designed in [17] leads to an issue when connecting a new node after a prolonged operation or when re-connecting the node to the network. Therefore, the author proposes the “Bell-X” mechanism, which has the dynamic EB transmission interval that iterates regularly. With the simulation result in which the Bell techniques with two compositions, technique with the fixed EB interval, and the RPL trickle timer-based EB transmission interval technique are compared, the author finds the point of compromise between network connection time and power consumption. [19], in the severely interfered environment, seeks to accomplish the quick synchronization utilizing the channel quality measurement. Using the RSSI-based channel quality measurement method, the channel in which the communication is easily available is selected, and this selection is used to perform the network formation. Before and after synchronization, nodes regularly estimate channel quality, resulting in additional energy consumption. [20] points out that the random-based EB scheduling method still bears the possibility of collision and proposes the collision-free advertisement scheduling (CFAS). Unlike other decentralized approaches, this method removes the collision. The simulation shows that it improves the average conjugation time while maintaining the energy consumption level.

### 2.3. Parallel Rendezvous Strategy

**Figure 3:**
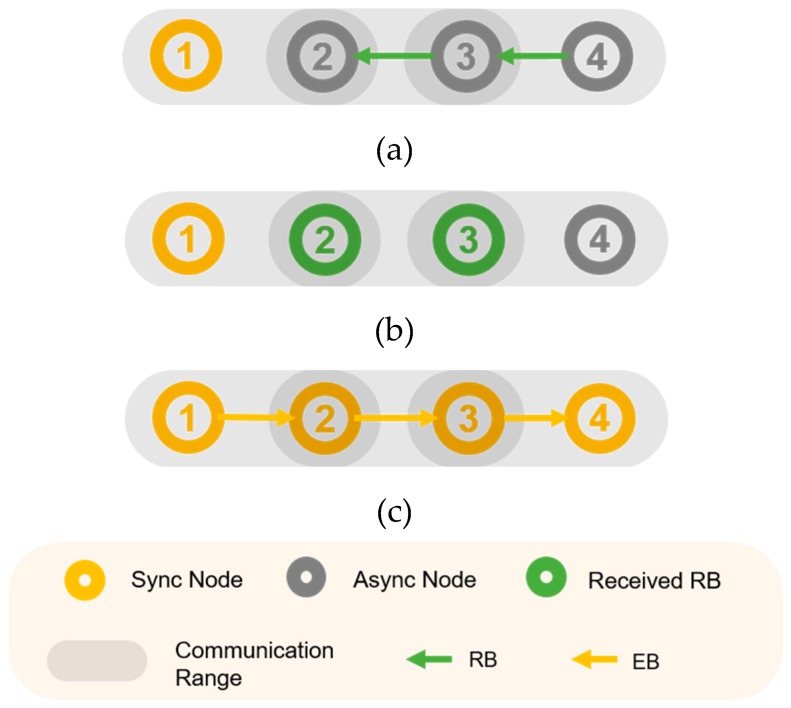
Example PRV–TSCH (**a**) Nodes 3 and 4 send RB (**b**) Nodes 2 and 3 receive RB (**c**) Fast synchronization using rendezvous packets.

In [8], the author proposed a parallel rendezvous-based algorithm called parallel rendezvous (PRV)–TSCH. The core of the PRV–TSCH scheme is to allow a node that is not yet synchronized to the network to send an RB containing the current channel and timing while searching for the channel, thus creating a rendezvous cluster prior to TSCH synchronization. That is, when a node receives a packet during an EB scan period, the node adds the source, channel index, and timing information to the rendezvous cluster’s neighbor. When the EB is received and synchronized to the network, the EB is transmitted to the scan channel through the channel information of the rendezvous cluster’s neighboring nodes. Before the synchronization, the nodes periodically transmit the RB, and they wait for a randomized time to prevent the collision between the RBs. Similarly to the EB-receiving mechanism, the channels of the transmitting side and receiving side may differ, and additional time is required to receive the RB and obtain the channel information. The node continuously informs its own channel information until it is synchronized, and the longer the synchronization time, it is more likely that nodes within the communications range obtain each other’s channel information. This allows the rendezvous nodes to be quickly synchronized to the TSCH network. In the three-hop linear topology of Figure 3, node 1 is the coordinator and demonstrates the approximate operation of PRV–TSCH. In step (a), nodes 3 and 4 transmit the RB before node 2 receives the EB. In step (b), when nodes 2 and 3 receive the RB, nodes 2 and 3 possess the channel hopping information and timing information of nodes 3 and 4, respectively. (c) When node 2 is synchronized, node 2 transmits the EB on the receiving channel of node 3, and when node 3 is synchronized, it transmits the EB packet on the receiving channel of node 4. Therefore, the time elapsed before all nodes synchronize to the network is shorter than that in the PS–TSCH.

## 3. The Novel Proposal: Distributed Radio Listening – TSCH

The distributed radio listening (DRL)–TSCH technique proposed in this study periodically transmits the enhanced rendezvous beacon (ERB) packets including channel information and destination node ID prior to TSCH network synchronization. Next, the node receiving the ERB calculates the channel of the node transmitting the ERB, and immediately transmits an ERB response packet to establish a rendezvous chain where each element is aware of the channel array information and indices of all the other elements in the chain. The rendezvous chain is defined as a two-way connection comprising a transmission and response that are each aware of the other’s channel index, channel array, and ID information before synchronization. When forming a rendezvous chain, the channels are subdivided. This action is carried out in order to increase the overall probability of EB reception in the nodes constituting the rendezvous chain, based on their channel information. The node ID uses the last two bytes of each node’s MAC address to identify and store the node and channel it has been assigned because of the division. Because the ERB response packet is transmitted through a single channel, the ERB response packet is transmitted immediately after the passage of some random amount of time to prevent packet collisions. When the ERB is received, the channel number of the sending node is calculated based on the timing information obtained at the receiving node.

### 3.1. 2-Way Strategy

**Figure 4:**
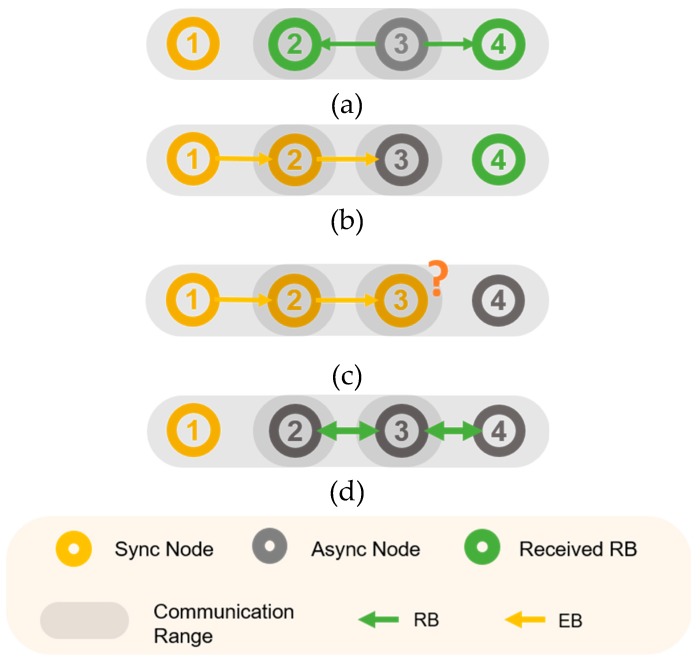
Two-way Rendezvous Benefits (**a**) Node 3 sends rendezvous packets, so nodes 2 and 4 receive (**b**) Node 2 is synchronized and sends EB to node 3’s channel (**c**) Node 3 does not know channel 4’s channel information (**d**) 2-way transmission rendezvous packet transmission strategy.

Packet transmission for parallel rendezvous on the TSCH network requires a formation time because of the channel mismatch between the transmission and reception, similar to the case with EB transmission and reception. When a node is synchronized to the network, only its rendezvous chain is quickly synchronized. Therefore, if nodes quickly exchange channel hopping information with one another before synchronization, the entire network can be synchronized quickly. Figure 4a–c show the three-hop TSCH network when only the rendezvous packet is transmitted periodically. In Figure 4a, node 2 and node 4 receive the rendezvous packet transmitted by node 3, and acquire the channel information of node 3. Because node 2 knows the channel information of node 3 after synchronization, node 2 transmits the EB to the listening channel of node 3 immediately after synchronization, so that node 3 can also be quickly synchronized. However, in Figure 4c, node 3 is not aware of the channel information of node 4. Node 4 is synchronized with the EB of Node 3 in the existing PS-TSCH scheme. Figure 4 shows the appearance when using a two-way connection. Similarly, when nodes 2 and 4 receive the rendezvous packet transmitted by node 3, node 3 knows the channel information of node 3, and immediately transmits the rendezvous packet to node 3. By establishing a rendezvous chain that is two-way, and ensuring that each chain element has knowledge of every other element’s channel information, the possibility of synchronization because of the rendezvous chain increases. This can contribute to rapid synchronization.

### 3.2. Distributed Radio Listening Through 2-way Connections

**Figure 5:**
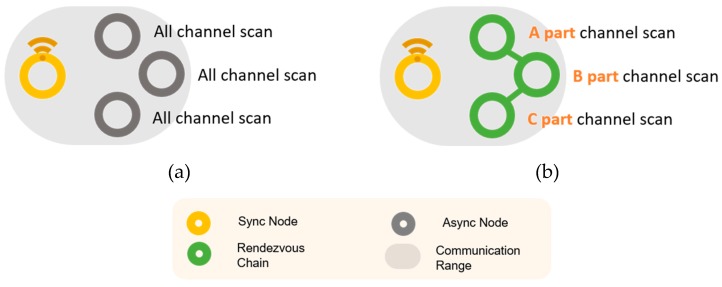
Distributed Radio Listening Through the Rendezvous Chain (**a**) Listen to all channels (**b**) Listen to the radio by dividing the channel.

Unlike linear topologies, in a mesh topology, EBs can be broadcast to multiple nodes and can be received from multiple synchronized nodes. In the topology of (a) of Figure 5, all existing channels are used to receive channels. If the receiving channel area is divided through the rendezvous chain as in (b), a faster synchronization speed can be expected. Therefore, in this study, we propose the DRL–TSCH scheme based on the two-way rendezvous chain. Prior to network synchronization, nodes periodically send ERB packets containing the node ID, channel array, and channel index. When receiving the ERB packet, we determine whether to partition the channel array into two using the received information of the channel array. The asynchronous node periodically selects a random channel on which to send the ERB. When another asynchronous node receives the ERB, it sends a response packet containing its node ID. Using this response, the channel can be divided between the two nodes such that both the nodes acquire channel information. The channel array of the node determines whether to partition the channel by comparing it with the channel array of the received ERB. If the channel array of the same node, or the channel array of any single node belongs to the channel array of another node, the channel is partitioned because another channel array is already being monitored by another node. If not, only the channel information is updated. If the node is not registered in the rendezvous chain, the ERB containing its ID is transmitted. A channel array can be subdivided into two separate channel arrays having an even and odd index. That is, when the ERB is received, the receiving side has an odd numbered channel of the channel array, and the remaining channel array is transmitted in an ERB response packet. The ERB response packet contains the ID of the node with which to partition the channel. “X-Ych” refers to the “Yth” array with the “X” number of channels; it describes multiple channel arrangements that occur due to the division of channel and do not overlap, and this explanation was added to the paper. If “i” is the indices of sixteen channel arrangement, it is the set of “i”s, the channel indices that are [ i mod (16 / X) == Y ]. For example, partitioning the channel arrangement 16 ch V [16] = {11, 12, 13, 14, 15, 16, 17, 18, 19, 20, 21, 22, 23, 24, 25, 26}, V [8] = {11, 13, 15, 17, 19, 21, 23, 25} and V [8] = {12, 14, 16, 18, 20, 22, 24, 26} produces 8-0 ch V and 8-1ch V. Subsequently, the 8-0 ch array can be divided into 4-0 ch and 4-1 ch arrays, and the 8-1 ch array can be divided into 4-2 ch and 4-3 ch arrays.

**Figure 6:**
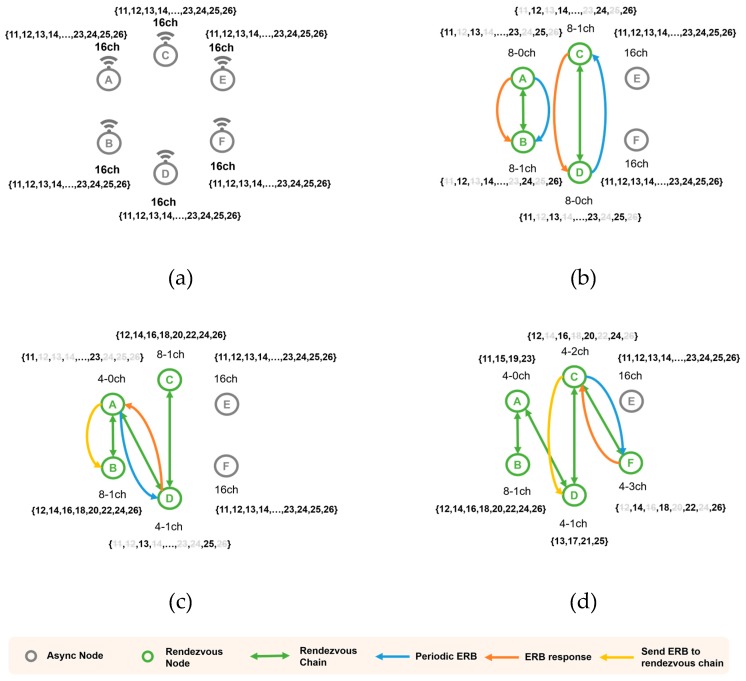
DRL-TSCH Operation Example (**a**) All nodes listen to 16 channels (**b**) A, B and C, D form rendezvous chain (**c**) A and D form the rendezvous chain (**d**) C and F form the rendezvous chain.

Figure 6 shows an example of the formation of the rendezvous chain. In Figure 6a, all nodes are listening to the signal by hopping 16 channels with 16 channel arrays. In Figure 6b, Nodes B and C receive an ERB packet and configure the 8-0 ch and 8-1 ch channel arrays, and transmit ERB response packets to A and D to form a rendezvous chain. Nodes A and D use the same channel array in (b) such that if ERB packets are exchanged, they can lead to the formation of the rendezvous chain as in (c). Furthermore, because A and D’s channel array information has changed, each node informs the neighboring chain nodes of the changed channel hopping information. During this process, the node can contribute to forming a rendezvous chain with another node. In Figure 6c, because the channel array of node C belongs to the channel array of node F in Figure 6d, if C and F exchange an ERB, the channel array can be partitioned. Channel arrays can be subdivided until there is only one channel. When an ERB packet is exchanged with a node with a single channel array, only the rendezvous chain is registered, but the channel array is not partitioned. Nodes in the rendezvous chain can be synchronized with a high probability, and when one node is synchronized, all nodes in the chain are quickly synchronized.

**Figure 7:**
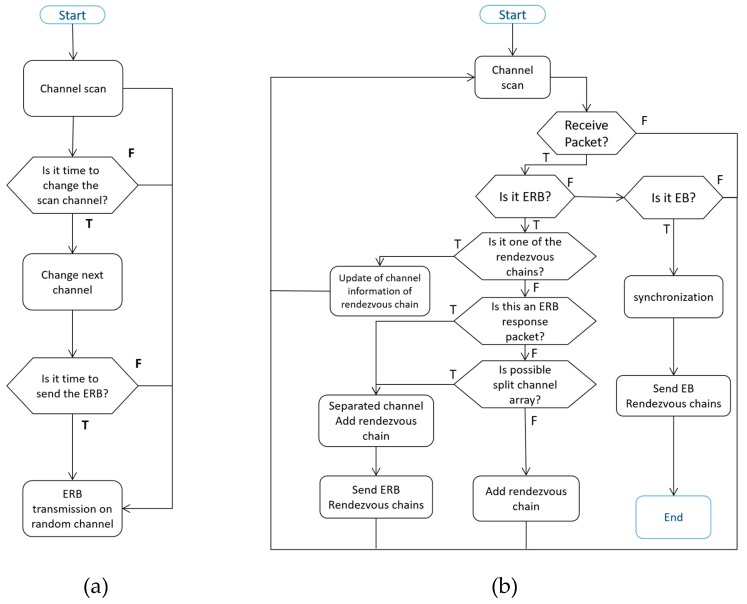
DRL–TSCH Flow Chart (**a**) Channel change and ERB packet transmission during channel scan (**b**) Operation when receiving ERB during channel scan.

Figure 7 shows the flow chart of DRL–TSCH before synchronization. Initially, Figure 7a shows a channel scan operation before synchronization. When it is time to change channels during the channel scan, the next channel is selected, and when it is time to send the ERB, the ERB is transmitted to a random channel. Figure 7b shows the operation when a packet is received during the channel scan. When the ERB packet is received, it checks whether the source node ID of the received ERB is already registered in the rendezvous chain. If a rendezvous chain has formed, the channel information of the node is updated, and the channel is scanned again. Next, the node checks to see if the received packet is a response packet from the ERB that it sent. If so, the node modifies the node’s own channel arrangement and registers the source of the ERB as the rendezvous chain. Next, the node checks if the channel can be split. If a split is possible, it splits the channel array and sends an ERB response.

In both cases, the ERB is sent to the nodes that make up the rendezvous chain. Finally, if the channel array length is unity or the received ERB channel array length is unity, it is registered as a rendezvous chain. When the node receives the EB in the scan state, it first synchronizes with the TSCH network following which it sequentially transmits the EB to the listening channel of the node registered in the rendezvous chain.

### 3.3. Hopping Channel Calculation Method

**Figure 8:**
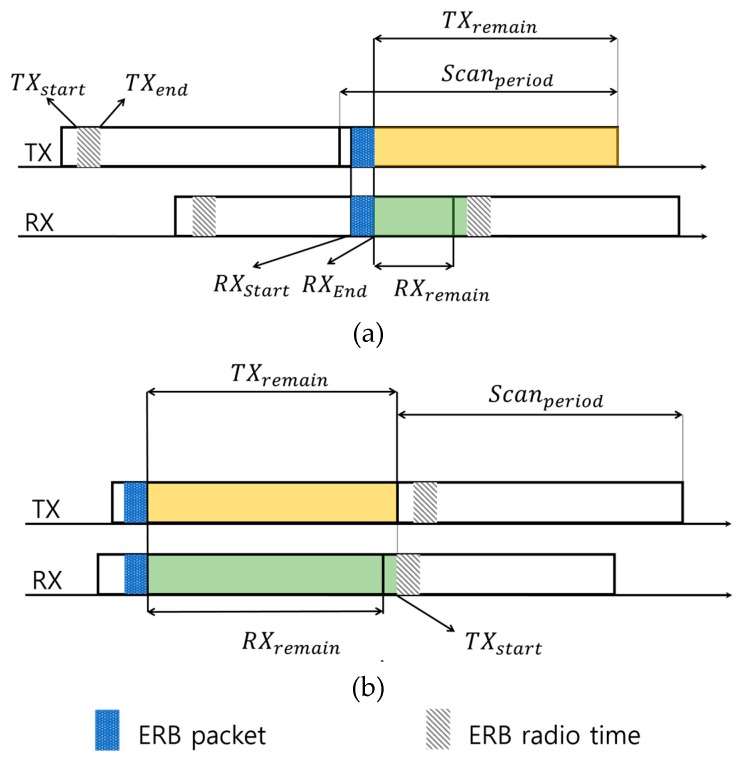
Two cases of ERB Receive Time (**a**) Receive timing should use previous channel index (**b**) Receive timing without changing channel index.

In the DRL–TSCH, the calculation of the current hopping channel of the rendezvous chain through the ERB packet is performed on the receiving node. The node transmits an ERB after a short offset for transmitting packets during the channel scan period on the current channel. Figure 8a,b displays the timing details of the transmission and reception of the ERB. The instant marked TX_start_ is the start time of ERB transmission, and TX_end_ marks the end of packet transmission; the time duration between these points is a fixed time. RX_start_ and RX_end_ are the beginning and end of the time interval during which the ERB is received. The ERB packet in the figure refers to the actual packet being sent or received. The ERB radio time refers to the time when an ERB packet should be transmitted during Scan_period_. When the RX node receives the ERB packet at the same time as (a), the TX channel does not change at the TX_start_ time of the next Scan_period_ of the RX. That is, the RX node shows that the channel index value of the ERB of the TX node should be recognized as the previous channel index value. Figure 8b shows that it should be used as the current channel index. After the ERB packet is received at RX, the TX channel is changed at the next Scan_period_ at TX_start_ time. In this case, because the channel is modified together when viewing TX from RX, the channel index is registered as is. That is, the channel calculation on the receiving side obtains the channel based on the received timing. The period resulting from the subtraction of TX_start_ from Scan_period_ is represented by TX_remain_, and is set as RX_remain_. This is the period obtained by subtracting RX_end_ from the Scan_period_. The channel index is determined by comparing RX_remain_ + TX_start_ with TX_remain_.

RX_remain_ + TX_start_ < TX_remain_ → index = index − 1, (2)

RX_remain_ + TX_start_ > = TX_remain_ → index (3)

The specified index is incremented every Scan_period_. An ERB containing a node ID or an EB meant for a particular node in a chain, is transmitted to the corresponding channel using the channel array information and the index value of the node to be transmitted.

### 3.4. ERB Packet Transmission

**Figure 9:**
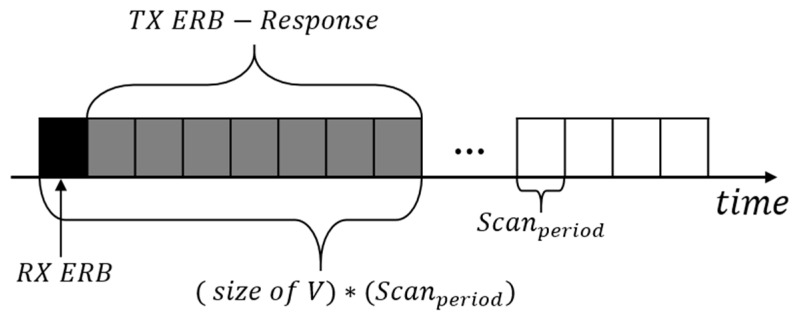
ERB Response Transmission Point.

The ERB is divided into packets for periodic transmission and packets for response. The ERB that transmits periodically, randomly transmits channels at each node. The node receiving this transmission checks whether it is already registered in the rendezvous chain; if so, it updates the channel information. If the node is not registered, it is registered as a neighbor of the rendezvous chain and decides whether to subdivide the channels by examining the channel arrangement. Periodic ERB packets can be received simultaneously by multiple nodes, and packet collisions can occur with response packets. In order to prevent this, the timing of the ERB response transmission is randomly transmitted to the “TX ERB-Response”, as shown in Figure 9. Also, the node sending the ERB modifies the channel array after (size of V) × (Scan_period_).

## 4. Experimental Evaluation

### 4.1. Experiment Setup

**Figure 10:**
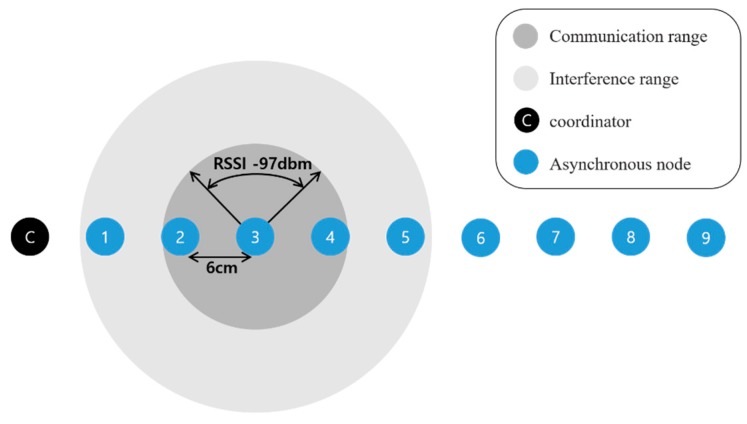
Nine-Hop Linear Topology.

**Figure 11:**
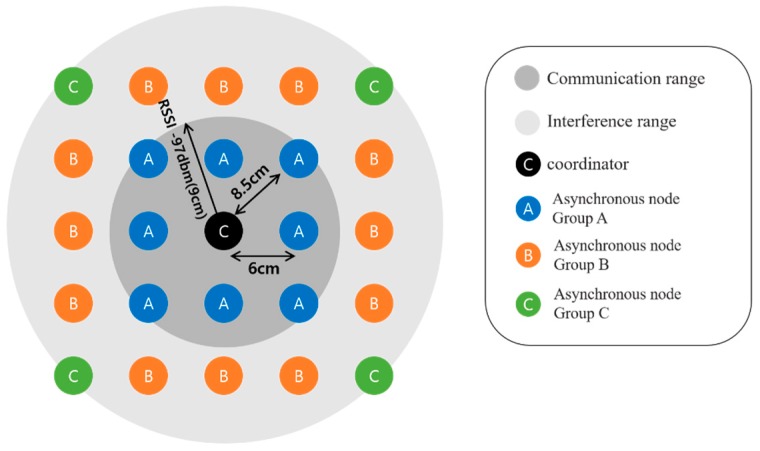
5 × 5 Grid Mesh Topology.

**Figure 12:**
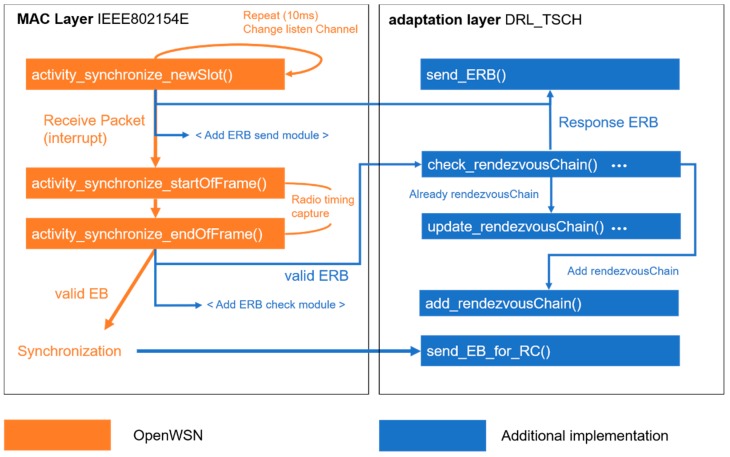
Implementation of the paper’s technique in OpenWSN.

The experiment was implemented based on the TSCH MAC Stack implemented in OpenWSN [21]. The communication module used was OpenMote-cc2538 [22], and the energy consumed was calculated according to the OpenMote-cc2538 specification. Table 1 shows the parameters used in the experiment. Experiments were carried out on a nine-hop linear topology (Figure 10) and a 25-figured grid mesh topology (Figure 11). In a nine-hop linear topology, each node was positioned at intervals such that the maximum RX sensitivity received signal strength indicator (RSSI) signal of the OpenMote-cc2538 was measurable. The communication range and interference ranges in Figure 10 and 11 shows that the node is in the middle of the range, and the remaining nodes have the same range. Each node can interfere with nodes located farther than the one-hop distance, and each node is synchronized sequentially by placing a coordinator node at the end of the topology. In the 5 × 5 grid mesh topology, a coordinator was placed in the middle, and the remaining nodes were placed at a one-hop distance based on the maximum RX sensitivity RSSI. In addition, we measured the nodes that had the same probability of receiving EB on the position of nodes by grouping them. The actual distance between each Hop is 6 cm and 8.5 cm (antenna hole), and the maximum is within the 9-cm range (approximately −97 dbm). Except for the antenna gain, performing the communication at a low output level reduces the signal strength, and it can physically reduce the environment of the topology in which the long-distance communication is guaranteed. There is no communication interference other than nodes utilized in the experiment, and the packet transmission speed is 250 kbps, the pre-existing default value. Figure 12 depicts the functions of nodes before synchronization that are additionally implemented in the OpenWSN. The nodes before synchronization are continuously listening to the channel using the “activity_synchronize_newSlot” function. The conventional synchronization method performs the synchronization using the radio interrupt upon receiving the EB packet, and the synchronization occurs when the EB is valid. In addition to this, the functions of the DRL–TSCH were added, and the module that transmits the ERB packets and the module that checks the ERB packets are used to exchange the channel information before the synchronization. Using the exchanged channel information, it is processed like the flowchart of Figure 7, and a rendezvous chain is constructed. In order to perform a quick synchronization to the rendezvous chain, which was established when the node synchronized, after synchronization, the node sends the EB sequentially to the channel of rendezvous chains. Group A is a node group one hop away from the coordinator and can be synchronized the fastest. And is in a position to broadcast the EB to the eight neighboring nodes. The nodes in Group B are in an environment where they can receive EBs from the five neighboring nodes and are placed two hops away from the coordinator. Group C can receive EBs from three neighboring nodes and is located two hops away from the coordinator. The experiment measured the synchronization time of each node and the number of rendezvous packet transmissions; the measurement was repeated a hundred times. The slot frame length and the shared slot presented in [7] are specified, and EB transmission is possible only in the shared slot. EB transmission is transmitted using the RV method of [13,14], which randomly selects a channel during transmission. The transmission probability is 33% to reduce the collision probability of EBs or rendezvous packets. The DRL–TSCH also has a 33% chance of sending rendezvous packets, but owing to the two-way transmission, it transmits more packets than the conventional PRV–TSCH. Thus, PRV–TSCH measures the transmission of rendezvous packets with two probabilities of 33% and 66%. This represents the relationship between the traffic of each rendezvous transmission scheme and the average synchronization time.

### 4.2. Experiment Result

#### 4.2.1. Nine-Hop Linear Topology

**Figure 13:**
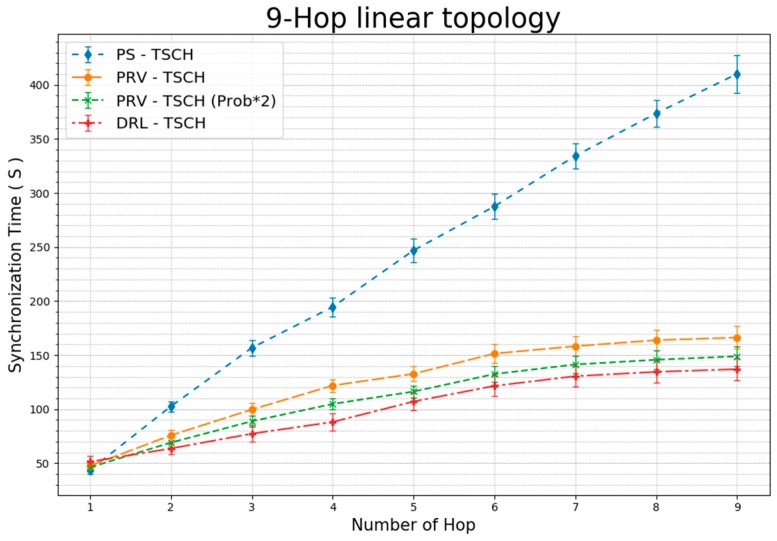
Average Synchronization Time for Each Hop Using 9-Hop Linear Topology (standard error).

Figure 13 shows the average TSCH network synchronization time for each hop. The one-hop node indicates that all the techniques are synchronized at similar times. In the PS–TSCH scheme, we observe that the synchronization time increases linearly with the number of hops. Since two-hop, the difference in synchronization time with other techniques has increased. The PRV–TSCH (Prob × 2) scheme, having twice the probability of rendezvous packet transmission, demonstrates a faster synchronization time than the PRV–TSCH strategy. DRL–TSCH represents synchronization results that are faster than those obtained using PRV–TSCH (Prob × 2). These results show that building a rendezvous chain through two-way transmission with the channel information of the received ERB rather than simply increasing the transmission probability can produce faster synchronization time. The DRL–TSCH demonstrates that the last node has approximately 67%, 18%, and 8% reduction in synchronization times when compared to the synchronization times obtained with other techniques.

**Figure 14:**
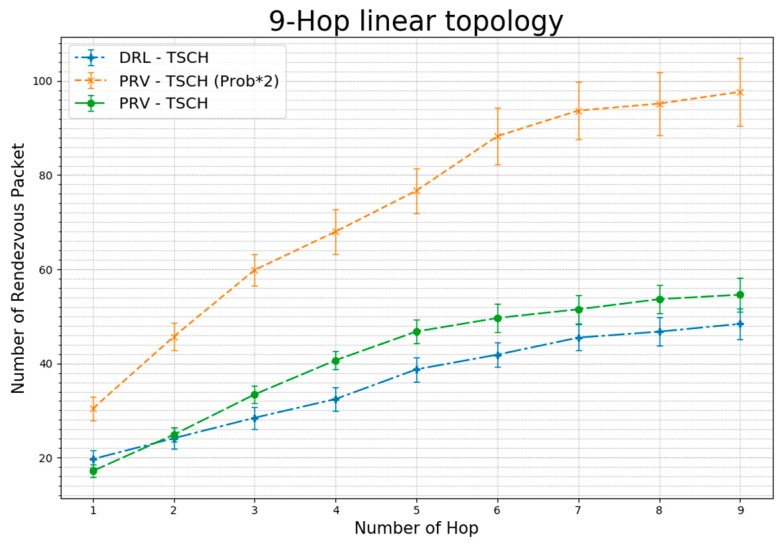
Average Number of Rendezvous Packets Transmitted per Hop Using 9-Hop Linear Topology (standard error).

Figure 14 shows the average number of rendezvous packets sent before synchronization. In the one-hop scheme, DRL–TSCH transmits more rendezvous packets than PRV–TSCH, but after three hops, the node demonstrates less rendezvous packet transmission until synchronization. This shows that in a linear topology, fast synchronization reduces traffic because of rendezvous packets. The PRV–TSCH (Prob × 2) transmits rendezvous packets with a 66% probability of transmission, which is higher than the 33% probability of PRV–TSCH. However, owing to the faster synchronization time, the number of rendezvous packet transmissions is less than twice the difference in packet transmission probability.

**Figure 15:**
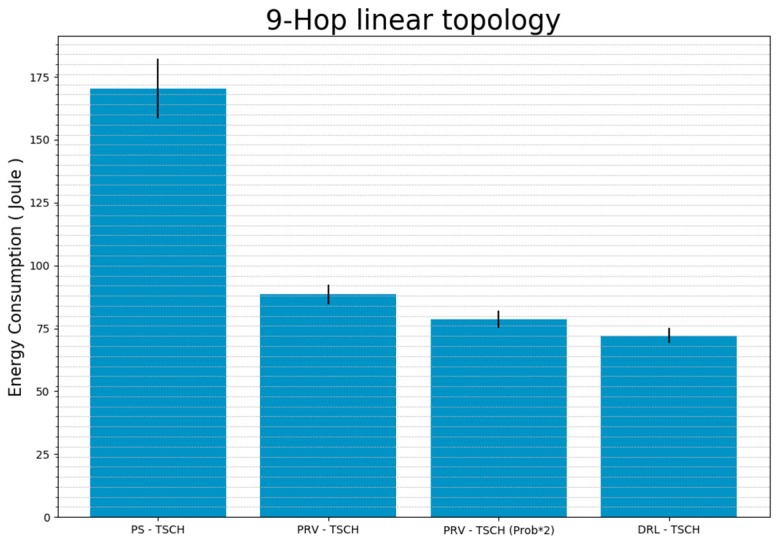
The Amount of Energy Consumed During Synchronization for Each Technique Using 9-Hop Linear Topology (standard error).

Figure 15 shows the sum of the total amount of energy used by the nodes before synchronization. The PS-TSCH used approximately 170 J of energy before synchronization, and the PRV–TSCH used approximately 88 J. This is approximately half of the energy consumed during PS–TSCH. PRV–TSCH (Prob × 2) that used approximately 78 J of energy, 12% less than that used by PRV–TSCH. The proposed technique, DRL–TSCH, consumed approximately 72 J and used the least amount of energy until all nodes were synchronized. Thus, DRL–TSCH consumed an energy amount reduced from PS–TSCH by 58%, 19%, and 8%.

#### 4.2.2. 5 × 5 Grid Mesh Topology

**Figure 16:**
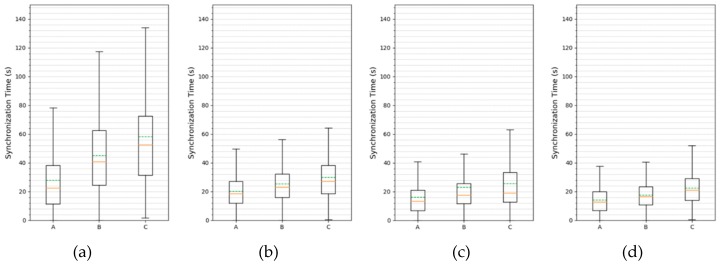
Distribution of Synchronization Time per Group Using 5 × 5 Mesh Topology (**a**) PS–TSCH (**b**) PRV–TSCH (Probability 33%) (**c**) PRV–TSCH (Probability 66%) (**d**) DRL–TSCH.

Figure 16 shows the box-plot of the synchronization distribution obtained using a 5 × 5 mesh topology. The solid line in the box represents the median and the dashed line represents the mean. In PS–TSCH, the width of the box and the beard are the largest. This shows that the distribution of synchronization time is wide and that the synchronization time for the worst case is much longer than in other schemes. The average synchronization time was also the longest in groups A, B, and C. The box sizes and whiskers from the box plots of the remaining schemes were similar, but they had the narrowest distribution in the DRL–TSCH, and the average synchronization time for each scheme was different for each group. Nodes in Group A can listen to the EB directly from the coordinator, resulting in the fastest synchronization time. Although Group B and Group C are at the same hop distance from the coordinator, the nodes in Group C demonstrate a slower synchronization time than the nodes in Group B because of the different number of neighbor nodes required to transmit EBs. PS–TSCH showed an average synchronization time of about 28, 45, and 58 seconds for each group. The average synchronization time of PRV–TSCH was approximately 20, 25, and 30 seconds, which are about 29%, 45%, and 49% less than PS–TSCH, respectively. The PRV–TSCH (Prob × 2) timings were about 16, 23, 25 seconds and decreased by about 20%, 8%, and 17%, respectively, when compared to the PRV–TSCH results. The proposed method, DRL–TSCH, showed the lowest average synchronization time, s, which was about 14, 17, and 22 seconds for each group. This is approximately 12%, 27%, and 12% less time than the synchronization time of the next fastest PRV–TSCH (Prob × 2) strategy.

**Figure 17:**
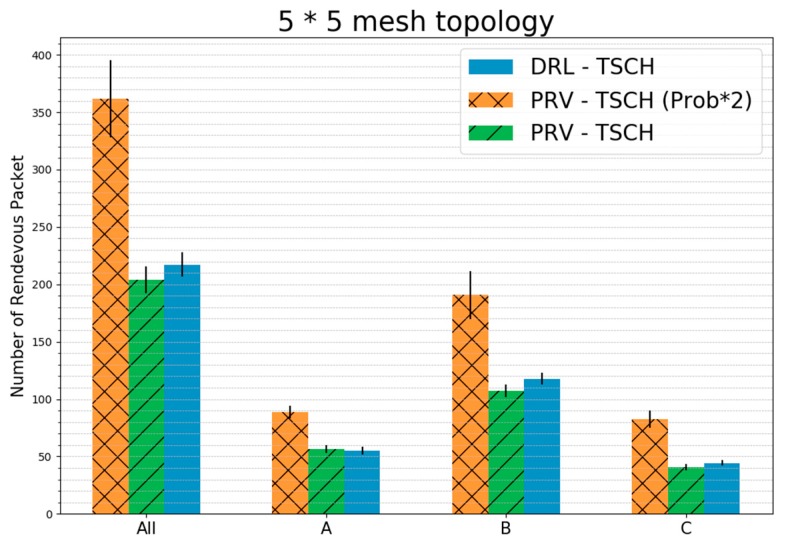
Average Rendezvous Packet Transmission before Synchronization in a 5 × 5 Mesh Topology (standard error).

Figure 17 shows the average rendezvous packet transmission for each group and all nodes in a 5 × 5 mesh topology. The PRV–TSCH (Prob × 2) generated 1.77 times the number of packets of the PRV–TSCH, yet resulted in lesser than twice the number of packet transmissions. This is because the synchronization rate of PRV–TSCH (Prob × 2) is faster than that of PRV–TSCH. In Group A, the number of packet transmissions of PRV–TSCH and DRL–TSCH was about the same. The number of packet transmissions in each group of DRL–TSCH was the smallest in the Group A compared to that of the other schemes. However, in the Group B and the Group C, more packets were transmitted despite their faster synchronization times when compared to that of PRV–TSCH. This is because more rendezvous packets have to be transmitted to partition the channels in the mesh topology than in the linear topology. Comparing the total number of packet transmissions, DRL–TSCH transmitted 7% more packets than PRV–TSCH, but the synchronization speed and energy consumption were much lower. In addition, the rendezvous packets transmitted were about 40% less than those in PRV–TSCH (Prob × 2), but were synchronized faster and with lower energy consumption.

**Figure 18:**
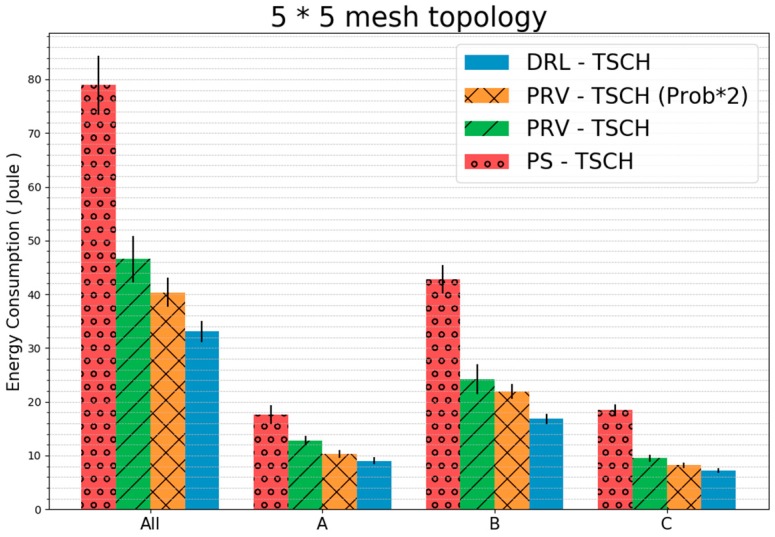
Energy Consumption During Synchronization in 5 × 5 Mesh Topology (standard error).

Figure 18 shows each group of the topology and the amount of energy used by all the nodes before synchronization. The energy consumption of all nodes of the PS–TSCH was about 78 J, which is consumed before synchronization when energy requirements are the most, and the remaining techniques consumed about 46, 40, and 33 J in the order shown in the figure. DRL–TSCH consumed the least energy of all groups. Because the difference in the node energy consumption of Group B, which has the largest number of nodes, is larger than that of the remaining groups A and C, the difference in the total energy consumption is larger than that of Group A and C. DRL–TSCH showed approximately 58%, 29%, and 18% decreases in total energy consumption. These numbers are presented in the order of energy consumption in comparison to the rest of the techniques.

## 5. Conclusions

In this study, we demonstrate faster synchronization speeds than those observed in existing schemes by subdividing the channel monitored in the TSCH protocol such that it hops up to 16 channels. Our strategy is based on the parallel rendezvous technique and on the construction of a rendezvous chain wherein each element is aware of the channel information of all the other chain elements. Experimental results demonstrate that in the linear topology, our scheme transmits the least number of packets before synchronization, has the fastest synchronization speed, and consumes 58% less energy than PS–TSCH. In the mesh topology, 7% more rendezvous packets were transmitted before synchronization than in the traditional PRV–TSCH; however, synchronization was 27% faster. DRL–TSCH facilitates immediate data collection with faster TSCH synchronization than conventional techniques, and reduces the energy consumed by RDC by 100% before synchronization. Especially the result depicted in Figure 13 shows that each technique produces around ten seconds of difference. Operating for ten seconds at the 100% RDC level leads to the same level of energy consumption of operating for 1000 seconds, or 16.6 minutes, at the 1% RDC level after the synchronization, and this difference cannot be disregarded in the situation that involves frequent re-establishment of the network.

In future research, we will study the method of reception of a single ACK by adding an ID to the EB packet to be transmitted to other rendezvous chains. This situation occurs when nodes in the rendezvous chain have to be synchronized in a crowded environment. When the EB transmitted to the rendezvous chain is not received, we will study the reliable synchronization of the nodes forming the rendezvous chain by using the method of retransmission after backoff.

## Figures and Tables

**Table 1:** Experiment Parameter Value.

Parameter	Value
run number	100
mote	Open Mote - cc2538
RX sensitivity	−97 dbm
TX Power	0 dbm
the number of channels	16
slot length / scan period	10 ms
slot frame length	101 slots
shared slot	0th slot
EB transmit probability	33%
rendezvous transmit probability	33% / 66%
data rate	250 kbps
antenna	none
